# Factors associated with overweight and abdominal obesity in Brazilian school-aged children: a comprehensive approach

**DOI:** 10.20945/2359-3997000000239

**Published:** 2020-03-30

**Authors:** Helen Carla Vieira Caixeta, Angelica Amorim Amato

**Affiliations:** 1 Laboratório de Farmacologia Molecular Faculdade de Ciências da Saúde Universidade de Brasília Brasília DF Brasil Laboratório de Farmacologia Molecular, Faculdade de Ciências da Saúde, Universidade de Brasília, Brasília, DF, Brasil

**Keywords:** Children, overweight, obesity, risk factors

## Abstract

**Objective:**

We investigated the association between demographic, socio-economic, perinatal, parental and lifestyle-related factors with general and abdominal obesity among prepubertal children aged 6 to 8 years in a Southeastern city of Brazil.

**Subjects and methods:**

A total of 486 children were randomly selected from public schools in the city of Patos de Minas, and examined to determine body mass index (BMI) and waist circumference (WC). Demographic, socio-economic, perinatal, parental and lifestyle-related data were obtained and assessed as independent risk factors for overweight/obesity and abdominal obesity, using multiple regression analysis.

**Results:**

Obesity/overweight (BMI percentile ≥ 85), seen in 19% of the children, was positively associated with low maternal education, being born small for gestational age, maternal BMI and screen time, whereas abdominal obesity (WC percentile > 90), seen in 9.9% of the children, was positively associated with maternal age and maternal BMI. When BMI and WC percentile were analyzed as continuous variables, birth by cesarean section, parental BMI, and lower sleep time were positively associated with BMI percentile, and birth by cesarean section, being born small for gestational age, and parental BMI were positively associated with WC percentile.

**Conclusion:**

Our findings suggest that the frequency of overweight and obesity in a city in the Southeastern region of Brazil is similar to the global frequency reported by the World Health Organization. We also found that many modifiable risk factors were associated with general and abdominal obesity, and these may possibly substantiate future strategies to prevent childhood obesity and its consequences in adult life.

## INTRODUCTION

Childhood obesity is currently one of the most serious global public health challenges, due to the alarming increase in its prevalence and associated morbidity and mortality (1). In addition to the short-term consequences of excess body weight, such as its negative impacts on academic achievement (2) and quality of life (3), children with obesity have an increased likelihood of being obese in adulthood and developing obesity complications, such as type 2 diabetes and cardiovascular diseases, at a younger age (4).

The obesity epidemic affects not only high-income countries but is growing even more rapidly in many low- and middle-income countries. Likewise, Latin America has one of the highest childhood overweight and obesity rates in the world, of around 20% (5). It is largely believed that the economic changes in Latin America over the last decades have favored increased consumption of calorically dense and low-nutrient foods, in addition to decreased physical activity, with a major impact on obesity epidemics. Notwithstanding, obesity is a complex and multifactorial disease, involving both modifiable and non-modifiable risk factors, whose relative contributions may vary on a regional basis (6). Given that obesity may be largely preventable, the acknowledgment of modifiable risk factors to ground regional policies addressing prevention is an urgent priority (7).

Brazil is an upper-middle income country in Latin America that has experienced a nutritional transition and dramatic increase in childhood obesity in urban areas, over the last decades (8). However, only few studies addressed the modifiable risk factors associated with increased body weight in children. We therefore aimed to comprehensively assess the association between known modifiable risk factors and the occurrence of overweight, obesity and abdominal obesity in children aged 6 to 8 years old in Patos de Minas, in the Southeastern region of Brazil.

## SUBJECTS AND METHODS

### Participants

This study protocol was approved by the Ethics Committee of the School of Health Sciences of the University of Brasilia, and written informed consent was obtained from all parents or guardians of the participants before enrollment. Prepubertal children aged 6 to 8 years were selected from public schools in the urban region of the City of Patos de Minas, at the Southeastern region of Brazil from April 2016 to August 2017. Exclusion criteria were cancer, untreated or inadequately treated hypothyroidism (serum thyrotropin levels above 10 mcUI/mL), unavailability of perinatal information (weight at birth), treatment with drugs associated with weight gain (systemic glucocorticoids or antipsychotics), or refusal to participate.

### Examinations

The parents underwent an interview to obtain demographic, perinatal and lifestyle-related data of children. Children underwent clinical assessment to obtain height and weight measurements to calculate body mass index (BMI), and waist circumference (WC). Nutritional status was assessed using BMI and the presence of abdominal obesity was assessed using WC. Overweight was defined as BMI percentile of 85 or higher, and abdominal obesity was defined as WC percentile of 90 or higher, according to CDC cutoff criteria (9,10). Severe obesity was defined as BMI percentile > 120% of the 95^th^ percentile (11).

### Statistical analysis

Sample size was estimated considering (i) that a total of 4,010 children aged 6 to 8 years were registered at public schools in Patos de Minas, in 2014, (ii) a 13.9% prevalence of childhood obesity, (iii) sampling error of 3%, (iv) two-sided type 1 error of 0.05, (v) and design effect of 1.5. A stratified two-step cluster sampling design with proportional allocation was used for subject selection. From a total of 27 public primary schools, 8 were randomly selected with probability proportional to the number of students. Then, within each of the 8 schools, the number of students was randomly selected also with probability proportional to the number of students.

BMI and WC (considered either as categorical – excess weight, BMI > p85; abdominal obesity, WC > p90 – or continuous variables) were the dependent variables. Independent variables were grouped into 4 blocks to perform a hierarchical stepwise regression as follows: (i) block 1, demographic variables (child’s age and sex, parents’ age and education level, mother’s marital status and profession, whether mother works at home or not, number of hours the mother works outside home, number of children at home, order of birth of the participant child, number of people at home, family income), (ii) block 2, perinatal variables (diabetes and/or hypertension during pregnancy, gestational age at birth, type of delivery, weight at birth, head circumference percentile at birth, breastfeeding, age at introduction of solid feeding), (iii) block 3, parents’ lifestyle-related variables (physical activity, comorbidities, BMI), and (iv) child’s lifestyle-related variables (daily caloric ingestion, daily number of meals, number of family shared meals, daily screen time, television at bedroom, mode of transport to school, physical activity, daily sleep time). Sample weights and the effect of complex sample design on the standard errors were treated using the survey command of STATA software version 10 (Stata Corp., College Station, Texas, USA).

The characteristics (demographic, socio-economic, perinatal, parental and behavioral) of children with normal BMI (5 to 85 percentiles) and increased BMI (≥ 85 percentile) were compared using unpaired Student t-test or qui-squared test. The correlation between BMI and WC percentiles was assessed using Spearman rank correlation analysis. Multiple Poisson regression model with robust variance was used to assess the prevalence ratio and 95% confidence interval of the association between excess body weight (BMI > p85) or abdominal obesity (WC > p90) and the independent variables. Multiple linear regression analysis was used to assess the linear coefficient (β coefficient) and 95% confidence interval of the association between BMI and WC and the independent variables. Within each hierarchical level, independent variables associated with dependent variables with a significance level (p) < 0.1 were considered adjustment factors for variables in the subsequent blocks. A tolerance indicator > 0.4 was considered the limit for the presence of multicollinearity between independent variables. A p value < 0.05 was considered statistically significant. Statistical analysis was conducted using STATA software, version 10.0.

## RESULTS

A total of 510 children were recruited; 23 children refused to participate and 1 child presented serum TSH level > 20 mcUI/mL and was referred to the pediatric endocrinology unit. The characteristics of the 486 children that were included in the study are shown in Table 1. Their mean age was 7 years, and there was a slight predominance of girls. A total of 48 (9.9%) and 44 (9.1%) of the included children were considered overweight or obese, respectively, and a 25 (5.1%) had severe obesity. The frequency of abdominal obesity was 9.9%. BMI and WC percentiles were highly correlated (Pearson r: 0.84; p < 0.0001). Children with normal BMI (5 to 85 percentiles) showed less total daily screen time, higher level of physical activity according to the perception of parents/guardians, and a higher frequency of shared family meals (lunch and dinner) than children with excess body weight (BMI ≥ 85 percentile). Moreover, maternal and paternal BMI were lower among children with normal body weight (Table 1).

**Table 1:** Characteristics of the children included in the study and their parents (n = 486)

Characteristic	BMI 5-85 percentile (n = 394)	BMI ≥ 85 percentile (n = 92)	p-value
Age – yr	7.0 ± 0.8	6.9 ± 0.8	0.50
Sex – no. (%)			
Female	207 (52.5)	43 (46.7)	0.32
Male	187 (47.5)	49 (53.3)	
WC percentile	46.8 ± 24.3	90.1 ± 9.4	**< 0.0001**
Order of birth – no (%)			
First	208 (52.8)	54 (58.7)	0.54
Second	120 (30.5)	26 (28.3)	
Third or further	66 (16.7)	12 (13.0)	
Birth weight (g)	3,143 ± 511	3,214 ± 568	0.24
Gestational age at birth			
Pre-term	44 (11.2)	12 (13.0)	0.76
Term	337 (85.5)	76 (82.6)	
Post-term	13 (3.3)	4 (4.4)	
Daily caloric intake (kcal)	1,591 ± 583.7	1,633 ± 693.8	0.55
Maternal age	35.3 ± 7.1	35.9 ± 7.4	0.50
Maternal BMI (kg/m^2^)	25.5 ± 5.1	27.9 ± 5.6	**< 0.0001**
Maternal marital status – no (%)			
Married/stable union	287 (73.8)	62 (67.4)	0.29
Single	72 (18.5)	19 (20.6)	
Divorced	28 (7.2)	9 (9.8)	
Widow	2 (0.5)	2 (2.2)	
Mother working outside home – no (%)	221 (56.8)	52 (56.5)	0.96
Maternal education – no. (%)			
Less than high school	125 (32.1)	31 (31.7)	0.21
High school graduate	414 (55.0)	42 (45.6)	
College degree	41 (10.6)	16 (17.4)	
Postgraduate degree	9 (2.3)	3 (3.3)	
Paternal age	38.8 ± 8.7	38.5 ± 8.3	0.79
Paternal BMI (kg/m^2^)	26.5 ± 4.3	27.3 ± 7.0	**0.045**
Paternal education – no (%)			
Less than high school	206 (55.4)	48 (53.9)	0.15
High school graduate	136 (36.6)	33 (37.1)	
College degree	27 (7.2)	7 (7.9)	
Postgraduate degree	3 (0.8)	1 (1.1)	
Monthly income – no. (%)^a^			
None	3 (0.8)	0 (0.0)	0.63
Up to 1	55 (14.0)	10 (10.9)	
1 to 3	207 (52.5)	48 (52.2)	
3 to 5	92 (23.3)	26 (28.2)	
5 to 10	31 (7.9)	8 (8.7)	
More than 10	6 (1.5)	0 (0.0)	
Number of siblings – no (%)			
0	110 (27.9)	31 (33.7)	0.27
1	185 (46.9)	46 (50.0)	
2	70 (17.8)	12 (13.0)	
3 or more	29 (7.4)	3 (3.3)	
Maternal smoking in pregnancy – no (%)	24 (6.1)	9 (9.8)	0.21
Gestational diabetes – no. (%)	24 (6.1)	8 (8.7)	0.36
Hypertension during pregnancy – no (%)	49 (12.4)	13 (14.1)	0.66
Birth by Cesarean section – no (%)	207 (52.5)	55 (59.8)	0.25
Breastfeeding – no (%)			
None	32 (8.1)	9 (9.8)	0.56
Less than 12 months	214 (54.3)	55 (59.8)	
12 to 24 months	99 (25.1)	18 (18.4)	
More than 24 months	49 (12.5)	11 (12.0)	
Meals together with family – no (%)			
Breakfast	173 (43.9)	31 (33.7)	0.07
Lunch	374 (94.9)	77 (83.7)	**0.0002**
Dinner	359 (91.1)	62 (67.4)	**< 0.0001**
Physical activity compared with peers’^b^			
Less	27 (6.9)	17 (18.5)	**< 0.0001**
Similar	157 (39.8)	44 (47.8)	
Greater	174 (44.2)	19 (20.7)	
Much greater	36 (9.1)	12 (13.0)	
Sleep duration (h/d)	9.5 ± 1.3	9.4 ± 1.2	0.64
<8	16 (4.1)	2 (2.2)	0.77
8-10	311 (78.9)	74 (80.4)	
11-12	65 (16.5)	15 (16.3)	
> 12	2 (0.5)	1 (1.1)	
Screen time (h/d)	3.9 ± 2.3	4.5 ± 2.6	**0.033**

^a^ Monthly income in minimal wages in 2017, in Brazil (approximately U$ 293).^ b^ According to the parent’s perception.

For maternal data n = 389.

AGA: adequate for gestational age; BMI: body mass index; LGA: large for gestational age; SGA: small for gestational age; WC: waist circumference.

The association between excess body weight (BMI percentile > 85) and demographic, socio-economic, perinatal, parental and lifestyle factors was examined by using multiple Poisson regression model with robust variance (Table 2). Higher maternal education (high versus primary school) was associated with a 20% reduction in the frequency of excess body weight, whereas being born small for gestational age increased the likelihood of having excess body weight by 2-fold. Maternal BMI and daily screen time were positively and significantly associated with excess body weight in children (Table 2). Abdominal obesity was significantly and positively associated with maternal age and BMI (Table 2).

**Table 2:** Association between socio-economic, demographic, perinatal, familial and lifestyle factors and the frequency of excess body weight (overweight and obesity) and abdominal obesity in 6 to 8-year-old children

	Overweight and obesity		Abdominal obesity	
	
	PR (95%CI)	*p*	PR (95% CI)	*p*
Demographic and socio-economic variables				
Maternal age	1.03 (0.99-1.06)	0.096	1.07 (1.00-1.15)	0.038
Paternal age			0.97 (0.94-1.00)	0.057
Child’s sex				
Female	1 (referent)	0.070		
Male	1.25 (0.98-1.59)			
Maternal education				
Primary school	1 (referent)	0.026		
High school	0.80 (0.67-0.97)			
Perinatal variables				
Birth weight				
SGA	2.16 (1.01-4.63)	0.049	1.75 (0.91-3.35)	0.081
AGA	1 (referent)		1 (referent)	
LGA	1.31 (0.48-3.58)	0.543	1.05 (0.28-3.91)	0.931
Familial variables				
Maternal BMI	1.05 (1.01-1.10)	0.019	1.05 (1.01-1.11)	0.032
Paternal BMI			1.07 (0.99-1.16)	0.073
Child’s lifestyle-related variables				
Screen time	1.09 (1.03-1.15)	0.012		

*p* value: multiple Poisson regression model with robust variance (hierarchical stepwise regression considering sequentially demographic and socio-economic variables, perinatal variables, familial variables and child’s lifestyle-related variables).

95% CI: 95% confidence interval; AGA: adequate for gestational age; BMI: body mass index; LGA: large for gestational age; PR: prevalence ratio; SGA: small for gestational age.

BMI and WC percentile were analyzed as continuous variables and their association with demographic, socio-economic, perinatal, parental and lifestyle factors was assessed by multiple linear regression analysis. We found that birth by Cesarean section, being born small for gestational age, and maternal BMI were significantly and positively associated with increased BMI percentile, whereas sleep time was negatively associated with BMI percentile (Table 3). Birth by Cesarean section, being born small for gestational age, and maternal and paternal BMI were positively and significantly associated with increased WC percentile (Table 3).

**Table 3:** Association between socio-economic, demographic, perinatal, familial and lifestyle factors and BMI and WC percentiles in 6 to 8-year-old children

	pBMI		pWC	
	
	β-coefficient (95%CI)	*p*	β-coefficient (95%CI)	*p*
Demographic and socio-economic variables				
Child’s age			-2.60 (-5.32, 0.12)	0.058
Perinatal variables				
Birth by Cesarean section	5.45 (0.47, 10.42)	0.014	6.75 (0.87, 12.64)	0.030
Birth weight				
SGA	19.31 (5.18, 33.45)		14.77 (2.32, 27.22)	0.026
AGA	Referent		Referent	
LGA	0.73 (-16.73, 18.20)	0.924	1.92 (-12.03, 15.88)	0.754
Familial variables				
Maternal BMI	1.32 (0.73, 1.91)	0.001	0.96 (0.35, 1.56)	0.007
Paternal BMI	1.04 (0.10, 1.97)	0.035	0.98 (0.27, 1.68)	0.014
Child’s lifestyle-related variables				
Sleep time	-1.62 (-2.84, -0.40)	0.016		

*p* value: multiple linear regression analysis (hierarchical stepwise regression considering sequentially demographic and socio-economic variables, perinatal variables, familial variables and child’s lifestyle-related variables.

95% CI: 95% confidence interval; AGA: adequate for gestational age; BMI: body mass index; LGA: large for gestational age; PR: prevalence ratio; SGA: small for gestational age.

## DISCUSSION

In this cross-sectional study, we found that the frequency of overweight and obesity were 9.9% and 9.1%, respectively, and the frequency of abdominal obesity was 9.9%, among overall healthy 6 to 8-year-old children. Using a multiple regression model, we found that lower maternal education level, being born small for gestational age, higher maternal BMI and higher screen time were associated with excess body weight, whereas maternal age and BMI were associated with abdominal obesity.

The children were recruited from randomly selected public schools in Patos de Minas, in the Southeastern region of Brazil. Within each school, children were selected randomly in proportion to the total number of 6 to 8-year-old students, so that the study sample would be representative. Moreover, the study participants were young, with a mean age of 7 years. Data from longitudinal studies indicate that the extent to which childhood obesity predicts adulthood obesity depends on a number of factors, such as age (12), the severity of obesity (12,13), BMI trajectory across childhood and adolescence (14), and parental BMI (15). Specifically, with respect to age, the likelihood of being an obese adult is higher for obese adolescents when compared with obese younger children (12). On the other hand, a cohort study following over 2,700 subjects from childhood (3 to 18 years) to adulthood (34 to 49 years) showed that those with a high BMI in adolescence but normal BMI in adulthood had lower BMI at the age of 6 years and a lower rate of BMI increase thereafter, suggesting that prevention of adult obesity should ideally begin at younger ages (14). Given these findings, and that the relative contribution of the various risk factors for obesity may plausibly differ across different ages, we viewed that investigating risk factors associated with obesity in young children would be of great value in understanding the risk factors for obesity at a regional perspective.

The frequency of excess body weight found in the current study is in concert with the global prevalence of overweight and obesity reported by the World Health Organization for children and adolescents aged 5 to 19 years in 2016 (1). It should be noted that we used the growth reference and criteria from the Center for Disease Control and Prevention, that have slightly higher cutoffs of BMI percentile/Z-score for overweight and obesity than those of the World Health Organization. Despite this, we found a slightly higher prevalence of obesity among children aged 6 to 8 years when compared with that described for children and adolescents aged 5 to 19 years (9.1% versus 6 to 8%) (1). The largest Brazilian survey involving 5 to 9-year-old children indicated that the prevalence of overweight and obesity were 19.2% and 14.3%, respectively, using WHO criteria (16). The divergence between these data and the findings reported herein is consistent with data from previous national studies indicating that the prevalence of obesity in Brazil varies widely according to the region of the country, ranging from 4.4% to 8.5% in the Northeast (17-19), and from 10.5% to 12.8% in the Southeast (20,21), most probably being related to economic factors. The frequency of obesity indicated by our findings is therefore in concert with data from the Southeast of Brazil.

Children with normal and increased BMI were similar with respect to many characteristics assessed herein, but in children with excess body weight (BMI percentile ≥ 85) showed higher daily screen time, less frequent family shared meals, lower physical activity levels, and higher parental BMI. In keeping with these findings, all these factors have been associated with childhood excess body weight in previous studies (22,23). Other characteristics associated with increased body weight among children in previous studies were not different between children with normal weight and excess weight in this study, highlighting that the factors associated with increased weight among children may be context specific, as has been previously discussed. Interestingly, we found that children with normal weight and overweight/obesity had similar daily energy intake, and energy intake was in agreement with the recommendations for daily caloric intake for boys and girls aged 6 to 8 years old (24). This is an intriguing finding that may be related to several non-mutually exclusive aspects. First, we estimated children’s energy intake using one 24-hour dietary recall informed by the parent, and a single 24-hour recall may be an inaccurate measure of energy intake (25). Although this may have reduced the accuracy of energy intake assessment in both normal weight and overweight children, underreporting of energy intake is more frequent among obese people, when compared to normal weight people (26,27). This may have selectively affected overweight children, since the presence of children’s overweight was associated with parent’s overweight in our study, and it is, therefore, plausible to speculate that parents may have underreported children’s energy intake. Second, given that children with excess body weight showed higher daily screen time and lower physical activity levels, it is plausible to hypothesize that decreased energy expenditure may have been a major factor affecting body weight. It is important to note, however, that this is essentially speculative, due to the cross-sectional design of the study and the lack of adjustment of this preliminary analysis for other factors affecting weight in children.

In order to gain more insights into the factors associated with increased body weight and WC, we comprehensively assessed demographic, socio-economic, perinatal, familial and lifestyle-related variables, using multiple regression analysis, considering BMI and WC as either categorical or continuous variables. When BMI was analyzed as a categorical variable, our findings indicated that lower maternal education level, being born small for gestational age, maternal BMI and screen time were positively associated with the presence of overweight/obesity. Small for gestational age status is associated with increased risk of obesity and metabolic disturbances not only in adulthood (28), but also earlier, during childhood itself (29). This association is most likely a causal one, and its biological basis possibly involves metabolic reprogramming, stemming from intrauterine growth restriction (28). Also in accordance with our findings, there is strong evidence that screen time is positively associated with excess body weight in children (30). Indeed, excess screen exposure affects several aspects of behavior that contribute to obesity (30).

Parental BMI is a strong predictor of children’s body weight, and this association seems to be more prominent with the mother’s weight status (15,31,32), in agreement with our findings. This possibly reflects both the genetic factors influencing weight status and the major role of parents in determining children’s lifestyle, behavior and environment that could affect the development of excess body weight (31). The association between parental education level and excess body weight in children was also addressed in previous studies, but the results varied widely. Both positive (33) and negative (34) associations were reported. Interestingly, negative associations, similarly to the one reported in the current study, were described in higher economic status countries, including Brazil (32), whereas positive associations were seen in lower economic status countries (32), suggesting the association of parental education and the likelihood of child overweight is complex and dependent upon the economic trajectory of the country.

When BMI was analyzed as a continuous variable, a positive association with both paternal and maternal BMI was found. This could suggest that, despite only maternal BMI could predict childhood overweight, both paternal and maternal BMI could predict an increased in body weight even among children with a normal BMI percentile (< 85), although the influence of maternal BMI was still of a greater magnitude in this analysis (linear regression coefficient of 1.32 for maternal BMI versus 1.04 for paternal BMI). Being born by cesarean section and less sleep time were also positively correlated with weight. Cesarean section was independently associated with childhood obesity in previous studies (35). The mechanisms underlying this association are not completely clear, but features of the infant gut microbiota of children born by cesarean versus vaginal delivery are implicated (35). Notably, we did not find an association between cesarean section and overweight/obesity, but only a positive association with BMI percentile. This possibly reflects the high rate of Cesarean section in both groups, of over 50% (normal and overweight). Lesser sleep time is also associated with increased likelihood of overweight in children (36). The lack of association with overweigh/obesity but linear association with BMI percentile possibly reflects the similar average sleep time between children with BMI percentile below and higher than 85.

Despite the finding that BMI and WC percentile were highly correlated, we also assessed the factors associated with intra-abdominal fat accumulation, assessed by WC measurement, in children. In adults, WC is well-known to be closely related to the risk of obesity complications, such as cardiovascular disease. Despite being more limited, there is also evidence that abdominal obesity in children is linked to increased cardiovascular risk (37). We found that maternal age and maternal BMI were positively associated with abdominal obesity in children. When WC was taken as a quantitative variable, birth by cesarean delivery, being born small for gestational weight and parental BMI were positively associated with WC values. Maternal age of 35 years or more at birth was associated with increased likelihood of childhood obesity (38). These data contrast to our findings, since herein maternal age of birth ranged from approximately 27 to 29 years and only the association with abdominal obesity but not general obesity was observed. The other factors associated with WC, either considered as a categorical or quantitative variable, were associated with obesity in other studies, as previously discussed, and our findings suggest they may also predict body fat distribution in children.

One of the main limitations of our work is its cross-sectional design, precluding the establishment of a causal association between obesity and the variables that were assessed. However, it is important to note that all the factors included herein were previously addressed in studies with a longitudinal design and found to be associated with the risk of obesity, at least in some populations. Another limitation of this study is that we could not adjust our analysis to genetic variants shown to influence weight status, and other factors related to obesity in children, such as gut microbiote composition. Therefore, we cannot rule out that the lack of adjustment of our results for these factors may have influenced our findings.

In conclusion, we found a frequency of overweight and obesity similar to other studies conducted in Latin America, of around 20%, and a frequency of abdominal obesity of 9.9%, among 6 to 8-year-old overall healthy children. Children with overweight and obesity showed significantly higher parental BMI, less frequent family shared meals, less physical activity and a greater screen time. In multiple regression analysis, general obesity was associated with lower maternal education levels, being born small for gestational age, maternal BMI and higher screen time, whereas abdominal obesity was associated with maternal age and maternal BMI. Since the modifiable risks factors for excess body weight in children may vary on a regional basis, these findings may help improve strategies to prevent childhood obesity.
